# Interest in Serving the Underserved: Role of Race, Gender, and Medical Specialty Plans

**DOI:** 10.1089/heq.2022.0064

**Published:** 2022-12-26

**Authors:** Rama A. Salhi, Ajith Dupati, John C. Burkhardt

**Affiliations:** ^1^ Department of Emergency Medicine, Massachusetts General Hospital, Boston, Massachusetts, USA.; ^2^ Department of Emergency Medicine, University of Michigan Health System, Ann Arbor, Michigan, USA.; ^3^ Department of Learning Health Sciences, University of Michigan Medical School, Ann Arbor, Michigan, USA.

**Keywords:** medical education, underserved patients, under-represented in medicine

## Abstract

**Introduction:**

Medical students often express their plans to care for medically underserved populations, but little is known about how this interest remains during medical school (MS). This study examined how self-reported interest in working with medically underserved communities may change during MS training based on several student characteristics.

**Methods:**

A secondary data analysis of all student records in the Electronic Residency Application Service (ERAS) from 2005 to 2010 is presented. Predictors included gender, under-represented in medicine (URiM) status, age, academic metrics, career interest, and medical specialty choice. Outcomes included interest in caring for medically underserved populations when entering MS, graduating MS, and graduating MS controlling for entering interest.

**Results:**

The total population included 6890 student records (49.5% women and 18.2% URiM). Women had a higher likelihood of being interested in practicing in underserved communities when entering and graduating MS (odds ratio [OR] 1.55, 95% confidence interval [CI] 1.37–1.77; OR 1.24, 95% CI 1.09–1.40). For all outcomes, URiM students had a higher likelihood of planning on a career with underserved populations compared with their non-URiM peers. Compared with Emergency Medicine, Internal Medicine/Pediatrics and Family Medicine had a higher likelihood of plans to work with underserved populations upon entering, graduating, and at graduation controlling for entering interest.

**Discussion:**

Gender, race, and specialty choice all had meaningful associations with a student's plans on practice in an underserved community. This study's findings can help support efforts to improve MS diversity nationally and drive study on cultural effects embedded within medical specialty identity.

## Introduction

The medical school (MS) selection process often attracts and selects for students interested in serving medically underserved and vulnerable populations.^1^ Time and again, disparities are identified in access to care, disease prevalence, morbidity, and mortality within these communities, which, for some, serves as the impetus to enter into a career in medicine.^2–8^ It is common for those interested in pursuing a career in medicine to identify these disparities as a call to action for entering the profession.

Persistent structural inequities can serve as a call to action for many students considering a career in the health professions. Whether the specific motivating disparity is most closely tied to race, socioeconomics, or geography, they are often related to a lived experience of being, or interacting with, a member of an underserved medical community. Such experiences have been correlated with an increased likelihood to choose to provide medical service to the underserved.^9–11^ However, we also know that differential educational access due to those same existing structural correlates results in students from some racial and ethnic groups remaining under-represented among MS graduates when compared with the broader population.

For students who identify with medically underserved communities in some manner, their premedical experiences or opportunities to work in underserved communities in MS may end up supporting their commitment upon completion of MS.^12^ As with other areas of learner persistence, we must also consider influences such as institutional and environmental factors in a student's maintained interest in working with underserved communities.^13^ Furthermore, maintenance of an identified underserved career plan may be differential by race, gender, or specialty.^14^

With respect to medical specialty choice, the development of an individual physician identity is intrinsically linked with how and where each student envisions their future practice of medicine and can change in concert with career plan reconsiderations.^15,16^ While any physician regardless of medical specialty type may dedicate their career to the underserved, specific medical specialties are traditionally linked with having a specific focus on providing care in these areas. While these designations are fluid and vary by perspective, historically primary care specialties^17,18^ and emergency medicine^19–22^ have explicitly made this role a part of their professional culture.

The process by which medical students cultivate and retain interest in serving underserved communities is poorly understood and likely multifaceted. Here, we aimed to better understand how a career interest in caring for underserved populations might change during MS training, specifically comparing differences associated with gender, race, and medical specialty choice. We used a series of secondary data analyses to explore the following hypotheses:

(1) Women entering MS will have a higher likelihood of reporting a planned career caring for underserved populations as compared with men.
(2) Under-represented in medicine (URiM) students will have a higher likelihood of reporting a planned career caring for underserved populations as compared with non-URiM students.
(3) Planned careers in caring for underserved populations will decrease in all groups but will decrease the least in URiM students compared with non-URiM students.
(4) The primary change observed will be one of attrition of relative decreased interest during MS with no group having a net increase interest over the same time period.
(5) Differences in planned careers caring for underserved populations will be stable and correlate with medical specialty choice at similar rates at the onset and completion of MS.

## Methods

### Study design

We secondarily analyzed data from students who applied for residency using Electronic Residency Application Service (ERAS) from 2005 through 2010. An important consideration of this dataset acknowledges that programs active in the National Resident Matching Program (NRMP) were not required to use ERAS before initiation of the all-in policy in 2012. However, the number of prematch offers made before 2012 was limited and largely involved international medical graduates.^23^ Outside of these circumstances, characteristics between ERAS and non-ERAS applicants have been noted to be similar.^24^ Individual records were linked and then deidentified by the Association of American Medical Colleges (AAMC) (database included a unique research identifier for each subject) from the following datasets to create a national-scale longitudinal database:

*Matriculating Student Questionnaire (MSQ):* questionnaire from the AAMC administered annually to U.S. matriculating medical students assessing topics including choosing medicine as a career and future career plan and interest.

*Graduation Questionnaire (GQ):* questionnaire from the AAMC to U.S. graduating medical students including specialty selection and future career plans and interests.

*AAMC Applicant Matriculant File (AAMF)*: dataset from the AAMC's centralized medical school application processing service including academic and demographic factors of applicants to MS.

*Electronic Residency Application Service (ERAS):* dataset of applicant data from the AAMC that is collected through their application to the National Residency Match Program.

*U.S. Medical Licensing Exam (USMLE):* The National Board of Medical Examiners allowed the inclusion of USMLE Step 1 and Step 2 CK.

Before any data analysis, institutional review was solicited, and the study approach was judged not to require additional regulation or assessment by the University of Michigan Institutional Review Board.

### Measurements

We selected predictor variables based on a review of the existing literature. The URiM variable represented a binary recoding of a self-reported racial/ethnic identity to either non-URiM (White or Asian students) or URiM student as defined by the AAMC. URiM status was defined in accordance with the current AAMC definition.^25^ In the case of multiple racial/ethnic identities, if any of these identities would have qualified the trainee as being from the URiM category they were classified in the URiM category.

Future medical specialty choices were grouped into one of nine categories (Internal Medicine and Subspecialties, General and Colorectal surgery, Other Surgical Subspecialties, Neurology and Psychiatry, Obstetrics/Gynecology, Pediatrics and Subspecialties, Internal Medicine/Pediatrics and Family Medicine, Other Outpatient-based Career, and Other Hospital-based Career) with complete details of each group's constituent specialties outlined in Table 1.

**Table 1.** Specialties Included in “Other Outpatient” and “Other Hospital-Based” Groupings

Other outpatient specialties	Other hospital-based specialties
Allergy and Immunology	Anesthesiology and subspecialties
Dermatology	Nuclear Medicine
Pain Medicine	Pathology and subspecialties
	Preventative Medicine or subspecialty
	Radiology
	Radiation Oncology
	Hospice and Palliative Care

A base comparison group was needed to complete the multinomial logistic reaction. We selected Emergency Medicine as the comparison group, given its purported role as the “social safety net for the uninsured” but its continued lower numbers of women and URiM applicants.^20,21,26–30^ Interest in taking care of underserved populations was derived from the response to the MSQ and GQ items: “Do you plan to care primarily for an underserved population?” In both cases, responses were then recoded as either “yes” or “no or undecided.”

Notably, reference categories within the model (e.g., male and non-URiM) were selected to highlight trends in the subgroups of interest (female and URiM students). In addition, metrics to control for the potential applicant competitiveness influencing decision making (including USMLE scores and grade point average [GPA]) and other career decision attitudinal responses to AAMC surveys were included to control for potential confounding factors.

### Analytic approach

We examined three outcomes: interest in caring for underserved populations at MS matriculation, interest in caring for underserved populations at MS graduation, and interest in caring for underserved populations at MS graduation while accounting for entering interest. Final models included specialty choice, gender, age, URiM status, GPA, MCAT, USMLE scores (steps 1 and 2), as well as other survey response items pertinent to factors contributing to specialty choice (listed in Table 4). Inclusion of variables in our final models was determined *a priori* based on the existing literature.

Students were only included in the final analysis if they had recorded responses for the variables under study. Given the significant rates of missingness inherent in the datasets^19^ and concern for potential bias introduced through this restriction, final models (Model 2, *n*=6890) are presented alongside less restricted models (Model 1, *n*=22,015). Logistic regression was used to fit these models, and calculate odds ratios (ORs) and confidence intervals (CIs).^31^ Analysis was performed using Stata 15.^32^

## Results

Among all the students included in the final model, 3455 (49.5%) were women and 1256 (18.2%) students identified as URiM (Table 2). Upon entering MS, the specialties with the highest number and percentage of student interest included Other Surgical Subspecialties (*n*=1457, 21.2%), Internal Medicine and Subspecialties (*n*=964, 14.0%), and Pediatrics and Subspecialties (*n*=952, 13.8%).

**Table 2.** Demographics of Respondents

Variable	Model 1		Model 2	
	Frequency	Percentage	Frequency	Percentage
Gender				
Male	11,323	51.43	3482	50.54
Female	10,692	48.57	3408	49.46
URiM status				
White and/or Asian	18,079	82.12	5634	81.77
URiM	3936	17.88	1256	18.23
MSQ specialty: interest starting MS				
Emergency Medicine	1831	8.32	549	7.97
Internal Medicine and Subspecialties	3147	14.29	964	13.99
General and Colorectal Surgery	2320	10.54	893	12.96
Other Surgical Subspecialties	4342	19.72	1457	21.15
Neurology and Psychiatry	1154	5.24	436	6.33
OB/Gyn	1149	5.22	362	5.25
Pediatrics and Subspecialties	3497	15.88	952	13.82
Internal Medicine/Pediatrics and Family Medicine	1947	8.84	336	4.88
Other Outpatient-based Career	925	4.20	321	4.66
Other Hospital-based Career	1703	7.74	620	9.00
GQ: medical specialty application categories				
Emergency Medicine	1802	8.19	571	8.29
Internal Medicine and Subspecialties	3339	15.17	1013	14.70
General and Colorectal Surgery	1830	8.31	634	9.20
Other Surgical Subspecialties	3270	14.85	935	13.57
Neurology and Psychiatry	1492	6.78	494	7.17
OB/Gyn	1598	7.26	522	7.58
Pediatrics and Subspecialties	2444	11.10	761	11.04
Internal Medicine/Pediatrics and Family Medicine	1719	7.81	451	6.55
Other Outpatient-based Career	653	2.97	196	2.84
Other Hospital-based Career	3868	17.57	1313	19.06
MSQ: planned practice with underserved populations				
No/unsure	16,787	76.25	5294	76.84
Yes	5228	23.75	1596	23.16
GQ: planned practice with underserved populations				
No/unsure	16,384	74.42	5036	73.09
Yes	5631	25.58	1854	26.91
*Total*	*22,015*	*100.00*	*6890*	*100.00*

GQ, Graduation Questionnaire; MS, medical school; MSQ, Matriculating School Questionnaire; OB/Gyn, Obstetrics/Gynecology; URiM, under-represented in medicine.

Upon graduation of MS, Other Hospital-based Careers (Appendix A1; *n*=1313, 19.1%), Internal Medicine and Subspecialties (*n*=1013, 14.7%), and Other Surgical Subspecialties (*n*=935, 13.6%) were the three most common specialties students intended to pursue. Entering MS, 1596 (23.2%) of students planned to pursue a career involving underserved populations. After graduation of MS, 1854 (26.9%) of the students planned on a career caring for underserved populations.

When entering and graduating from MS, women were more likely than men to express their interest in a career involving underserved populations (OR 1.55, CI 1.37–1.77; OR 1.24, CI 1.09–1.40). However, when controlling for effects of entering interest in a career with underserved populations, the difference in likelihood between women and men's graduating interest was mitigated (OR 1.09, CI 0.95–1.24).

**FIG. 1.**
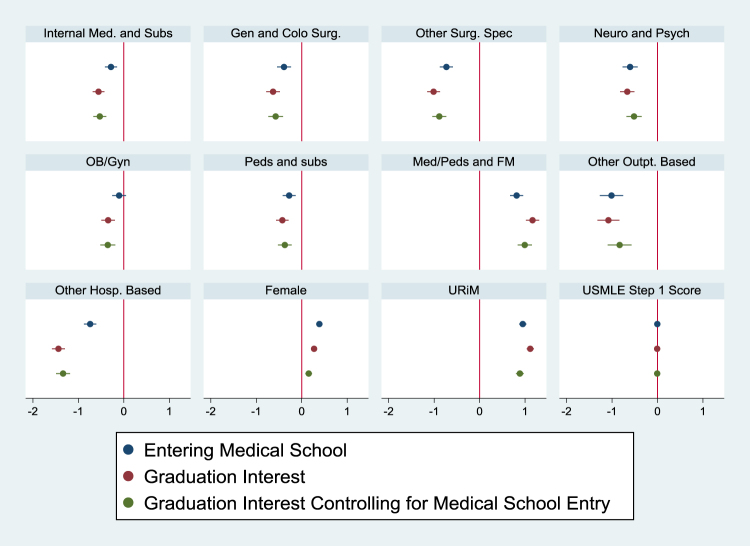
Trends in expressed interest in working with underserved communities, beta estimates with corresponding CIs (Model 1). CIs, confidence intervals.

**FIG. 2.**
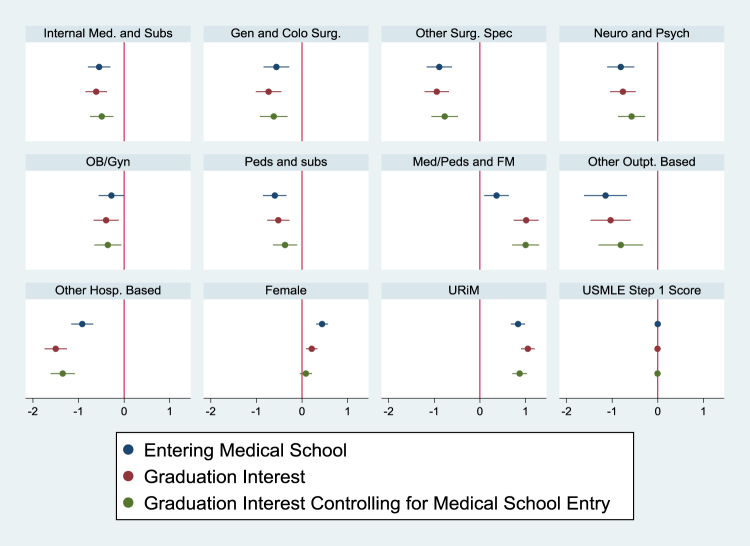
Trends in expressed interest in working with underserved communities, beta estimates with corresponding CIs (Model 2).

Relatedly, upon both entering and graduating MS, URiM students had increased odds of reporting interest in working with underserved communities (OR 2.31, CI 1.98–2.70; OR 2.86, CI 2.46–3.33, respectively) compared with non-URiM students. The increased likelihood of URiM students planning on a career in service to underserved medical populations upon graduation (when compared with non-URiM peers) was still significantly higher even when controlling for their increased likelihood of this career plan at the time of entering MS (OR 2.39, CI 2.037–2.811). Similar trends were seen in both Models 1 and 2 (Table 3, Fig. 1; Table 4, Fig. 2).

**Table 3.** Model 1—Examining Adjusted Associations with Outcomes of Interest

Variables	Entering interest		Graduating interest		Graduating controlling for entering interest	
	OR (SE)	95% CI	OR (SE)	95% CI	OR (SE)	95% CI
Medical specialties^a^						
Internal Medicine and Subspecialties	0.75^^*^^ (0.051)	0.660–0.861	0.57^^*^^ (0.038)	0.504–0.653	0.59^^*^^ (0.043)	0.512–0.680
General and Colorectal Surgery	0.68^^*^^ (0.053)	0.581–0.790	0.53^^*^^ (0.041)	0.458–0.619	0.56^^*^^ (0.047)	0.479–0.663
Other Surgical Subspecialties	0.48^^*^^ (0.035)	0.417–0.557	0.36^^*^^ (0.026)	0.315–0.419	0.41^^*^^ (0.033)	0.353–0.481
Neurology and Psychiatry	0.55^^*^^ (0.047)	0.463–0.647	0.51^^*^^ (0.041)	0.439–0.602	0.60^^*^^ (0.051)	0.504–0.706
OB/Gyn	0.90 (0.071)	0.774–1.052	0.71^^*^^ (0.054)	0.610–0.822	0.70^^*^^ (0.059)	0.596–0.829
Pediatrics and Subspecialties	0.76^^*^^ (0.055)	0.658–0.876	0.65^^*^^ (0.046)	0.569–0.750	0.69^^*^^ (0.053)	0.593–0.801
Medicine/Pediatrics and Family Medicine	2.26^^*^^ (0.167)	1.955–2.613	3.21^^*^^ (0.237)	2.774–3.708	2.70^^*^^ (0.216)	2.310–3.159
Outpatient based	0.36^^*^^ (0.048)	0.281–0.470	0.34^^*^^ (0.042)	0.267–0.432	0.43^^*^^ (0.058)	0.334–0.566
Other Hospital based	0.48^^*^^ (0.033)	0.416–0.547	0.24^^*^^ (0.017)	0.206–0.274	0.26^^*^^ (0.021)	0.225–0.307
Women	1.48^^*^^ (0.053)	1.375–1.582	1.31^^*^^ (0.047)	1.225–1.408	1.17^^*^^ (0.044)	1.083–1.257
Age, year	1.06^^*^^ (0.005)	1.049–1.070	1.03^^*^^ (0.005)	1.023–1.044	1.01^^*^^ (0.006)	1.003–1.025
URiM	2.59^^*^^ (0.104)	2.390–2.799	3.05^^*^^ (0.124)	2.817–3.302	2.43^^*^^ (0.107)	2.227–2.647
USMLE Step 1 Score^b^	1.00^^*^^ (0.001)	0.995–0.998	0.99^^*^^ (0.001)	0.991–0.995	0.99^^*^^ (0.001)	0.992–0.996
Medical School Debt	1.01^^*^^ (0.002)	1.004–1.013	1.00 (0.002)	0.997–1.006	1.00 (0.002)	0.993–1.003
Entering Interest Control					5.86^^*^^ (0.221)	5.447–6.313
Constant	0.11^^*^^ (0.030)	0.0652–0.190	0.68 (0.184)	0.396–1.154	0.66 (0.195)	0.367–1.174
Observations	22,021	22,021	22,166	22,166	22,015	22,015

^*^ Significant at *p*<0.05.

^a^ Comparison Group: Emergency Medicine, Men; Robust SEs in parentheses.

^b^ (per one-point increase).

OR, odds ratio; SE, standard error; USMLE, U.S. Medical Licensing Exam.

With respect to medical specialty, Internal Medicine and Subspecialties, General and Colorectal Surgery, Other Surgical Subspecialties, Neurology and Psychiatry, Pediatrics and Subspecialties, and Other Outpatient and Hospital-based Careers were less likely to show an interest in underserved populations compared with Emergency Medicine. This finding persisted upon entering, graduating, and graduating MS when controlling for entering interest (Table 4; Fig. 2). On the contrary, Internal Medicine/Pediatrics and Family Medicine had the highest likelihood of planning on caring for the underserved across entering interest, graduating interest, and graduating interest when controlling for entering interest.

**Table 4.** Model 2—Examining Adjusted Associations with Outcomes of Interest

Variables	Entering interest		Graduating interest		Graduating controlling for entering interest	
	OR (SE)	95% CI	OR (SE)	95% CI	OR (SE)	95% CI
Medical specialties^a^						
Internal Medicine and Subspecialties	0.58^b^ (0.073)	0.452–0.740	0.54^b^ (0.065)	0.428–0.686	0.61^b^ (0.080)	0.474–0.792
General and Colorectal Surgery	0.57^b^ (0.082)	0.430–0.756	0.48^b^ (0.069)	0.363–0.637	0.54^b^ (0.083)	0.398–0.729
Other Surgical Subspecialties	0.41^b^ (0.058)	0.311–0.541	0.39^b^ (0.053)	0.297–0.507	0.46^b^ (0.068)	0.346–0.618
Neurology and Psychiatry	0.44^b^ (0.067)	0.330–0.598	0.47^b^ (0.066)	0.351–0.615	0.56^b^ (0.085)	0.419–0.756
OB/Gyn	0.76 (0.108)	0.573–1.000	0.67 (0.094)	0.511–0.887	0.70^b^ (0.106)	0.520–0.941
Pediatrics and Subspecialties	0.55^b^ (0.073)	0.426–0.713	0.59^b^ (0.074)	0.466–0.759	0.69^b^ (0.093)	0.529–0.898
Med/Pediatrics and Family Med	1.44^b^ (0.197)	1.102–1.885	2.76^b^ (0.386)	2.097–3.631	2.73^b^ (0.412)	2.029–3.666
Other Outpatient-based Career	0.32^b^ (0.076)	0.199–0.509	0.36^b^ (0.080)	0.229–0.552	0.44^b^ (0.111)	0.272–0.725
Other Hospital-based Career	0.40^b^ (0.049)	0.314–0.507	0.22^b^ (0.028)	0.175–0.285	0.26^b^ (0.035)	0.199–0.339
Women	1.55^b^ (0.103)	1.365–1.769	1.24^b^ (0.079)	1.091–1.403	1.09 (0.073)	0.953–1.242
Age, year	1.03^b^ (0.012)	1.009–1.055	1.05^b^ (0.011)	1.026–1.070	1.04^b^ (0.012)	1.017–1.062
URiM	2.31^b^ (0.184)	1.976–2.699	2.86^b^ (0.220)	2.463–3.327	2.39^b^ (0.197)	2.037–2.811
Cumulative Grade Point Average^c^	1.00^b^ (0.001)	0.995–1.000	1.00 (0.001)	0.996–1.000	1.00 (0.001)	0.997–1.001
MCAT Total Score^c^	0.99 (0.009)	0.976–1.011	0.98^b^ (0.009)	0.959–0.992	0.97^b^ (0.009)	0.956–0.992
USMLE Step 1 Score^c^	1.00 (0.003)	0.994–1.004	0.99^b^ (0.003)	0.989–0.999	0.99^b^ (0.003)	0.989–0.999
USMLE Step 2 CK score^c^	1.00 (0.002)	0.996–1.004	1.00 (0.002)	0.996–1.004	1.00 (0.002)	0.996–1.005
Work–life balance	0.89^b^ (0.032)	0.828–0.954	0.98 (0.035)	0.912–1.049	1.02 (0.039)	0.944–1.095
Specialty personality	0.95 (0.062)	0.834–1.077	0.98 (0.063)	0.868–1.117	1.01 (0.067)	0.883–1.148
Specialty competitiveness	0.94 (0.032)	0.879–1.006	0.95 (0.033)	0.890–1.020	0.96 (0.035)	0.898–1.037
Advice from mentor	1.07^b^ (0.033)	1.004–1.132	1.11^a^ (0.035)	1.042–1.178	1.09^a^ (0.036)	1.024–1.167
Medical school debt in US$10,000	1.01 (0.004)	0.998–1.013	0.99 (0.004)	0.987–1.002	0.99^b^ (0.004)	0.984–1.000
Number of publications (per publication)	0.98^b^ (0.007)	0.963–0.992	0.98^b^ (0.007)	0.971–0.997	0.99 (0.007)	0.977–1.004
Research experience	1.01 (0.019)	0.973–1.048	1.00 (0.019)	0.963–1.040	0.99 (0.021)	0.952–1.033
Awarded AOA before application	0.95 (0.094)	0.787–1.158	1.04 (0.102)	0.859–1.261	1.07 (0.112)	0.871–1.314
Confidence in specialty choice	0.96 (0.052)	0.868–1.072	0.98 (0.055)	0.878–1.094	0.99 (0.060)	0.883–1.119
Entering interest control					5.03^a,b^ (0.338)	4.411–5.740
Constant	1.03 (0.779)	0.232–4.542	2.00 (1.495)	0.464–8.650	1.08 (0.856)	0.228–5.107
Observations	6891	6891	6906	6906	6890	6890

^a^ Comparison Group: Emergency Medicine, men; robust SEs in parentheses.

^b^ Significant at *p*<0.05.

^c^ (per one-point increase).

AOA, alpha omega alpha; CK, clinical knowledge; MCAT, Medical college admission test.

## Discussion

We observed several trends in medical student interest in working with underserved patient populations. As hypothesized, women entering MS were significantly more likely to express their interest in working with underserved communities as compared with men (OR 1.55, CI 1.37–1.77). This association also held true among URiM students who were found to be over twice as likely (OR 2.31, CI 1.98–2.69) as non-URiM students to express their interest in a career working in medically underserved communities upon entering MS.

To better examine the impact of MS on students' decisions and to expound on previously demonstrated associations^15^ we examined these trends again upon graduating from MS. To date, a large portion of available literature focuses on specialty selection and the association with URiM status. Here too, both women and URiM students were more likely, than men and non-URiM students, respectively, to express their intent in working with underserved communities.

Notably, and building on these previously demonstrated associations, the observed impact appears to be somewhat diminished in magnitude among women as compared with entrance into MS (OR 1.24, CI 1.09–1.40). As with specialty selection, there is a similar suggestion of a combined prematriculation and MS impact on students' final decisions.^33,34^ This is consistent with the current literature in demonstrating the impact of MS on students' interest in working with the underserved.^35^

To better understand how the time spent in MS may have changed some students' career plans, we completed analyses controlling for entering interests. In these comparisons, URiM students were persistently more likely to express their interest in practicing among underserved communities, although the effect size was smaller than upon entering. A similar trend toward the null was seen among women after controlling for entering interest, who were equally likely to express interest in working with underserved communities as men. Trends toward the null suggest a missed opportunity in MS toward increasing students' interest in serving underserved communities.

When examining these trends broken down by students' clinical interests, we found that, as compared with Emergency Medicine, the majority of medical specialty choices were associated with a lower likelihood of planning for a career caring for underserved medical populations. This held true upon entering MS, graduating MS, and at graduation controlling for entering interest.

In contrast to trends toward the null seen among women, URiM students, or other specialties, Family Medicine, and combined Internal Medicine/Pediatrics showed an increased interest in working with underserved communities. Overall, students who chose to specialize in Family Medicine or Internal Medicine/Pediatrics were >2.5 times more probable to plan on a career in the service of underserved populations upon graduation than their Emergency Medicine-bound peers. This may be in part related to Family Medicine trends toward working in underserved communities as a specialty, providing medical students with exposure and fostering similar interests for those who choose to pursue the career.^36,37^

### Health equity implications

Interventions targeted to narrow disparities in both patient outcomes and representative enrollment into MS should consider a multifaceted approach to increase their likelihood of success. In our analysis, we observed persistent differences associated with racial background and planned medical specialty choice. These associations were true even after controlling for students' entering interests indicating an additional effect occurring between matriculation and graduation. Possible mediators of that effect include cultural norms, values, and beliefs perpetuated and modeled during the medical education process.

As observed previously, even when controlling for entering interest in working with underserved patient population, URiM students are still more likely to express this career plan at the time of graduation compared with their non-URiM peers. This could indicate that their experiences during MS act as an additional reinforcement of this interest or choice.

Alternatively, this could also indicate that non-URiM students' experiences in MS result in a decreased probability of developing a career interest in working with the underserved. Some combination is also possible instead of only one group having a meaningful attitudinal change in response to the same stimulus. Future research, including both prospective quantitative and in-depth qualitative analyses, could shed further light on these associations and their underlying cases.

### Limitations

While providing important insights, this study does have limitations. Our work includes a secondary data analysis of records collected by the AAMC and USMLE. Although often used as a powerful tool in informing gaps, both survey data and secondary data analyses have inherent limitations. As the data were not primarily collected, the variables available for analysis were limited and not specifically designed to address our research question. To address this limitation, additional variables were included from other related data sources.

Another potential limitation is the reliance on survey data regarding specific student attitudes. While, when possible, we used actual independently obtained records from the primary data holder (such as Step scores from USMLE and not reported scores from surveys), however this was not possible in all cases. As such, our accuracy of survey factors may be limited by nonresponse and potentially by social desirability bias. Furthermore, as missingness was relatively limited in the datasets utilized, it is unlikely for this to have meaningfully skewed the results of the presented data.

## Conclusion

In our study, gender, racial background, and medical specialty choice all had initial and persistent correlations with a student's plans toward a career working with underserved patients. Of specific importance was the fact that attitudes upon entering MS were inadequate to explain significantly lower likelihoods of some students' plans to care for the underserved. This is consistent with an effect during MS, which may be modifiable by medical educators. Finally, this work once again highlights the importance of supporting URiM students both before and after MS matriculation, as well as broadly fostering and nurturing interest in caring for underserved communities throughout the MS pipeline.

## Data Sharing

AAMC data can be accessed at https://www.aamc.org/data-reports. NBME data can be requested at https://www.nbme.org/services/request-data

## Abbreviations Used

AAMC: Association of American Medical Colleges
AOA: alpha omega alpha
CIs: confidence intervals
CK: clinical knowledge
ERAS: Electronic Residency Application Service
GPA: grade point average
GQ: Graduation Questionnaire
MCAT: medical college admission test
MS: medical school
MSQ: Matriculating School Questionnaire
NBME: National Board of Medical Examiners
NRMP: National Resident Matching Program
OB/Gyn: Obstetrics/Gynecology
ORs: odds ratios
SE: standard error
URiM: under-represented in medicine
USMLE: U.S. Medical Licensing Exam

## Appendix

### Appendix A1.

**Other outpatient specialties:**

Allergy and Immunology

Dermatology

Pain Medicine

**Other Hospital Based:**

Anesthesiology and subspecialties

Nuclear Medicine

Pathology and subspecialties

Preventative Medicine or subspecialty

Radiology

Radiation Oncology

Hospice and Palliative Care
